# Brain monoamine oxidase A in seasonal affective disorder and treatment with bright light therapy

**DOI:** 10.1038/s41398-018-0227-2

**Published:** 2018-09-21

**Authors:** Marie Spies, Gregory M. James, Chrysoula Vraka, Cécile Philippe, Marius Hienert, Gregor Gryglewski, Arkadiusz Komorowski, Alexander Kautzky, Leo Silberbauer, Verena Pichler, Georg S. Kranz, Lukas Nics, Theresa Balber, Pia Baldinger-Melich, Thomas Vanicek, Benjamin Spurny, Edda Winkler-Pjrek, Wolfgang Wadsak, Markus Mitterhauser, Marcus Hacker, Siegfried Kasper, Rupert Lanzenberger, Dietmar Winkler

**Affiliations:** 1 Department of Psychiatry and Psychotherapy, Medical University of Vienna, Vienna, Austria; 2 Department of Biomedical Imaging and Image-guided Therapy, Division of Nuclear Medicine, Medical University of Vienna, Vienna, Austria; 3 Laboratory of Neuropsychology, The University of Hong Kong, Pokfulam, Hong Kong; 4 The State Key Laboratory of Brain and Cognitive Sciences, The University of Hong Kong, Pokfulam, Hong Kong; 5 CBmed GmbH, Graz, Austria; 6 Ludwig Boltzmann Institute for Applied Diagnostics, Vienna, Austria

## Abstract

Increased cerebral monoamine oxidase A (MAO-A) levels have been shown in non-seasonal depression using positron emission tomography (PET). Seasonal affective disorder (SAD) is a sub-form of major depressive disorder and is typically treated with bright light therapy (BLT). The serotonergic system is affected by season and light. Hence, this study aims to assess the relevance of brain MAO-A levels to the pathophysiology and treatment of SAD. Changes to cerebral MAO-A distribution (1) in SAD in comparison to healthy controls (HC), (2) after treatment with BLT and (3) between the seasons, were investigated in 24 patients with SAD and 27 HC using [^11^C]harmine PET. PET scans were performed in fall/winter before and after 3 weeks of placebo-controlled BLT, as well as in spring/summer. Cerebral MAO-A distribution volume (V_T_, an index of MAO-A density) did not differ between patients and HC at any of the three time-points. However, MAO-A V_T_ decreased from fall/winter to spring/summer in the HC group (*F*_1, 187.84_ = 4.79, *p* < 0.050), while SAD showed no change. In addition, BLT, but not placebo, resulted in a significant reduction in MAO-A V_T_ (*F*_1, 208.92_ = 25.96, *p* < 0.001). This is the first study to demonstrate an influence of BLT on human cerebral MAO-A levels in vivo. Furthermore, we show that SAD may lack seasonal dynamics in brain MAO-A levels. The lack of a cross-sectional difference between patients and HC, in contrast to studies in non-seasonal depression, may be due to the milder symptoms typically shown by patients with SAD.

## Introduction

Seasonal affective disorder (SAD) is characterized by decreased mood and drive in the fall and winter months, followed by spontaneous remission in spring and summer^1^. Two to three percent of the population suffers from clinically manifest SAD^2^. Bright light therapy (BLT) is well accepted as the gold standard treatment of this disorder^3^.

The central contribution of serotonergic changes to depressive pathophysiology, irrespective of seasonality, has been thoroughly demonstrated. Along this line, increased cerebral monoamine oxidase A (MAO-A) levels are considered a central finding in non-seasonal major depressive disorder (MDD)^4^. MAO-A is a mitochondrial enzyme that is primarily responsible for degradation of serotonin (5-HT) in the brain and is of utmost importance for homeostasis of cerebral 5-HT levels^5^. Increased MAO-A in MDD is therefore in accordance with the monoamine theory of depressive pathophysiology.

As is the case in MDD^6^, serotonergic changes are also thought to underlie development of SAD. Numerous molecular components of the serotonergic system are influenced by season and light, including the 5-HT_1A_-receptor^7^ and the serotonin transporter (SERT)^8–11^. Furthermore, seasonal regulation of SERT expression was shown to be altered in SAD^12,13^. In addition, the efficacy of MAO-A inhibitors in SAD^14^ emphasizes that changes to MAO-A may play a role in the disorder’s pathophysiology. However, changes to cerebral MAO-A distribution have yet to be investigated in patients with SAD.

This study thus investigates whether brain MAO-A distribution (1) differs between patients with SAD and healthy controls (HC), (2) is sensitive to BLT and (3) changes between fall/winter and spring/summer. In addition, the association between SAD symptom severity and MAO-A was investigated. For this purpose [^11^C]harmine (7-[^11^C]methoxy-1-methyl-9*H*-[3,4-*b*]indole) positron emission tomography (PET)^4,15,16^ was used and MAO-A distribution volume (V_T_), an index auf MAO-A density, was calculated. [^11^C]harmine is a reversible MAO-A inhibitor, is selective for the MAO-A isoenzyme^15^ and its metabolites do not pass the blood–brain barrier^17^. It can therefore be considered the gold standard of currently available MAO-A radioligands for cerebral PET imaging^4^.

Along the lines of the hyposerotonergic hypothesis of depression^4,6^ and in reflection of the influence of season and light on other molecules regulating serotonergic neurotransmission, higher MAO-A V_T_ in fall/winter, reduction after BLT and normalization in spring/summer was expected in SAD.

## Materials and methods

### Subjects

Twenty four patients suffering from unipolar SAD and 27 HC were included in this study. Sample size calculation was performed using G*Power based on effect sizes estimated from Meyer et al.^18^ using matched pairs *t*-test with a calculated effect size of 0.7, alpha 0.05 and power 0.95. Diagnosis was determined using the Diagnostic and Statistical Manual of Mental Disorders (4th ed., text rev.; DSM-IV-TR; American Psychiatric Association, 2000) and the Structured Clinical Interview for DSM-IV for Axis I disorders (SCID-I for DSM-IV). The SCID-I was utilized to exclude Axis-I comorbidities in patients and any Axis-I disorders in HC. All patients exhibited a Seasonal Problem Score ≥ 2 together with a Global Seasonality Score ≥ 10 on the Seasonal Pattern Assessment Questionnaire (SPAQ)^19^. An experienced physician administered the SCID and instructed subjects on how to complete the SPAQ. Patients were free from all psychopharmacologic treatment and BLT within the last 6 months as well as all pharmacologic substances during study participation, including until after PET3. Severe internal or neurologic disorders were excluded using medical history and physical examination, electrocardiography and blood tests. Urine tests were used at screening to exclude current drug abuse, current smoking^20^ and pregnancy. Breastfeeding women were excluded. During study participation, subjects were instructed to follow their typical routines, so as to limit bias resulting from lifestyle changes. Recruitment of SAD patients and HC was performed via the outpatient clinic at the Department of Psychiatry and Psychotherapy at the Medical University of Vienna and dedicated message boards at the Medical University of Vienna. All participants provided written informed consent and received financial reimbursement for participation. This study was approved by the Ethics Committee of the Medical University of Vienna and performed according to the Declaration of Helsinki.

### Experimental design

A first PET scan (PET1) was performed in fall/winter, before start of BLT. Patients and controls were then randomized to active BLT or placebo in a double blind manner (SAD-BLT, SAD-placebo, HC-BLT, HC-placebo). Subjects and study physicians were blinded. Both SAD and HC received placebo-controlled BLT in order to assess if resulting MAO-A changes were an SAD-specific treatment effect, or rather a general neurobiological reaction to BLT. The second scan (PET2) was performed in fall/winter after 3 weeks of daily BLT/placebo. The third PET (PET3) was performed in spring/summer (mean difference PET1–PET2 ± SD = 34.70 ± 12.38 days, mean difference PET2–PET3 ± SD = 155.91 ± 40.18 days, excluding anticyclic subjects). Six of 27 HC were recruited in an anticyclic manner and underwent PET3 in spring/summer first, followed by PET1 and PET2 before and after BLT/placebo in the consecutive fall/winter. A structural MRI scan was performed at any point during study participation for co-registration of PET data. The fall/winter and spring/summer seasons spanned from October 8^th^ to April 22^nd^ and April 17^th^ to September 17^th^, respectively.

### Bright light therapy

BLT was performed over the course of 3 weeks using an artificial white light source (PhysioLight LD220 by Davita Medical Products, Kranenburg, Germany) with full-spectrum 10,000 lux light intensity (at a distance of 50 cm from the light source). Treatment was administered daily, for 30 min, before noon. This treatment scheme was based on previous studies^3,21^. Three weeks were chosen as the treatment time-frame because changes to other serotonergic molecules have been shown after a similar time-period of BLT^10^. To promote compliance, therapy was addressed at following visits. Placebo control consisted of a non-biologically active light source^22^. The placebo lamp was the same in size and shape as the therapeutic device, however the light intensity was reduced to <400 lux at 50 cm using a transparent filter within the device.

### Imaging methods

#### PET

[^11^C]harmine (7-[^11^C]methoxy-1-methyl-9*H*-[3,4-*b*]indole) synthesis and quality control was performed according to procedures described by Philippe et al.^23^ All PET data was acquired using a GE Advance full-ring scanner at the PET Center at the Medical University of Vienna and in accordance with the center’s standard protocols. For tissue attenuation correction, a transmission scan of 5 min was carried out with ^68^GE rod sources. An intravenous bolus of [^11^C]harmine (4.6 MBq/kg body weight) was administered simultaneously with the start of PET. Dynamic PET was performed in 3D mode and reconstructed in 35 transaxial section volumes (128 × 128 matrix) with an iterative filtered backprojection algorithm (FORE-ITER) with a spatial resolution of 4.36 mm full-width at half maximum next to the center of the field of view. Fifty one successive time frames (12 frames of 5 s, 6 frames of 10 s, 3 frames of 20 s, 6 frames of 30 s, 9 frames of 1 min and 15 frames of 5 min) were collected, resulting in a total acquisition time of 90 min, optimized for modeling of [^11^C]harmine. Arterial blood samples for [^11^C]harmine quantification were drawn automatically for the first 10 min and manually at standardized intervals after [^11^C]harmine injection^15^.

#### MRI

All subjects underwent one T1-weighted structural magnetic resonance imaging (MRI) scan for co-registration of PET data. Structural co-registration was performed using SPM12 (Wellcome Trust Centre for Neuroimaging, London, United Kingdom; http://www.fil.ion.ucl.ac.uk/spm/). MRI was performed using a 3 T PRISMA MR Scanner (Siemens Medical, Erlangen, Germany, 1 × 1 mm voxel size, 1.1 mm slice thickness, 200 slices) or a 3 T Achieva MR Scanner (Philipps, Best, Netherlands, 0.47 × 0.47 mm voxel size, 0.88 mm slice thickness, 180 slices).

### Quantification of monoamine oxidase A

Manual arterial blood samples were processed according to Ginovart et al^15^. A gamma counter, cross-calibrated with the PET scanner and the arterial blood sampling system, was used to calculate radioactivity concentrations. Quantification was carried out using PMOD 3.509 (PMOD Technologies Ltd., Zurich, Switzerland; www.pmod.com). Whole blood activity was multiplied with plasma-to-whole blood ratio and the fraction of intact radioligand in the plasma to obtain the final arterial input function (AIF). V_T_ of specifically bound radioligand was quantified using a constrained two-tissue compartment model (2TCM) by coupling K1/k2 over several regions of interest (ROIs)^15^. Constrained 2TCM was used because this model resulted in more stable standard error of the fit across regions in this data set than the unconstrained method. For quantification, regions were taken from an automated anatomical labeling-based atlas (amygdala and hippocampal complex, cingulate cortex, frontal, temporal, parietal and occipital area, insula, midbrain, striatum, thalamus and cerebellar gray matter)^24^. Next, voxel-wise quantification was carried out using Logan plot, whereby the AIF and the time activity curve of the thalamus, a stable high uptake region, served as input. From the resulting distribution images, all regional V_T_ except midbrain were extracted using the Harvard-Oxford probabilistic atlas and averaged for both hemispheres. ROIs for statistical analyses were selected based on the investigation by Meyer et al.^4^ and included the frontal and temporal pole, anterior and posterior cingulate gyrus, caudate, putamen, thalamus, hippocampus and midbrain. A global ROI consisting of the above-mentioned ROIs and weighted for ROI size was calculated for regression analyses.

To demonstrate the consistency of the quantification methods used, and because V_T_ may be underestimated by Logan plot, V_T_ values calculated for coupling of K1/k2 and V_T_ values used for statistical analyses were compared. Furthermore, repeated measures analyses of variance (ANOVA) were performed with K1/k2 values and free fraction of tracer in blood to demonstrate that changes shown in V_T_ can be attributed to changes in k3/k4 (Supplement).

### Psychometric assessment

For assessment of depressive symptom severity at PET1, PET2 and PET3, the Beck Depression Inventory (BDI)^25^ was used for self-rating and the 24-item Hamilton Depression Rating Scale (HAMD) was used for external rating^26^. An experienced physician administered the HAMD and instructed subjects on how to complete the BDI. All psychological scores including those performed at the screening visit (SCID, SPAQ) were performed in the clinical setting using paper-based report forms.

### Statistical analyses

In order to assess changes to symptom severity (BDI, HAMD), repeated measures ANOVA with measurement (PET1, PET2, PET3) as within subject factor, therapy arm (BLT, placebo) and group (SAD, HC) as between subject factors and symptom severity scores (BDI, HAMD) as dependent variables were performed.

Regression analyses were performed in order to assess if certain clinical variables had a significant influence on MAO-A V_T_. Variables showing significant effects on MAO-A V_T_ were then included as covariates in subsequent mixed model analyses of MAO-A V_T_ data. Regression analyses with age, sex, perimenopausal status and symptom severity (BDI, HAMD) as independent variables and global MAO-A V_T_ as the dependent variable were performed at PET1, PET2 and PET3. Perimenopausal status was defined as female sex and age ≥ 41 years.

In order to assess changes to MAO-A V_T_, linear mixed model analyses using measurement (PET1, PET2, PET3), therapy arm (BLT, placebo), group (SAD, HC) and region (9 ROIs) as fixed factors, subject as random factor and MAO-A V_T_ as dependent variable were performed. Clinical variables showing significant effects on MAO-A V_T_ in the regression analyses described above were included as covariates.

Furthermore, initial mixed model analyses were repeated without the three subjects (1 SAD, 2 HC) who were scanned between 17^th^ and 22^nd^ of April to exclude the relevance of overlapping scan dates between the seasons.

In an exploratory analysis, mixed models were repeated without covariates. In addition, an exploratory mixed model analysis with therapy arm (BLT, placebo), group (SAD, HC) and region (9 ROIs) as fixed factors, subject as random factor and the difference between MAO-A V_T_ at PET1 and PET3 as dependent variable, was performed to assess if seasonal change in MAO A V_T_ differed between SAD and HC or therapy arms.

Prerequisites for statistical tests were evaluated (i.e., normal distribution, homogeneity of variance) and violations were accounted for (i.e., choice of covariance structure). For repeated measures ANOVA of symptom severity scores (BDI, HAMD) and mixed model analyses of MAO-A V_T_, post hoc models were corrected using Fisher’s least significant difference procedure in accordance with the closed test principle if factors showed a significant interaction in the primary model. Post-hoc models were declared nonsignificant if the *p* value of the primary interaction effect was nonsignificant, but carried out without further correction in case of a significant primary interaction effect. For all regression analyses, multiple testing was corrected for using the Holm–Bonferroni method, unless otherwise stated.

## Results

PET data was acquired in 26 SAD and 36 HC and could be analyzed in 24 SAD (mean age ± SD = 34.21 ± 10.57 years, 8 male) and 27 HC (33.52 ± 10.57 years, 11 male). Of the 24 SAD who underwent PET1, 21 and 18 underwent PET2 and PET3, respectively. Of the 27 HC included in this study, PET data was available at PET1 and PET2 for 26 and at PET3 for 24 (Table 1).

**Table 1:** Clinical variables

	SAD		HC
**BDI (PET1)**			
BLT	17.08 ± 5.63^*^	^**^	0.83 ± 1.40
Placebo	11.91 ± 5.86		1.38 ± 2.10
**BDI (PET2)**			
BLT	11.17 ± 7.27^***^	^**^	0.92 ± 1.78
Placebo	7.75 ± 5.65		0.21 ± 0.58***
**BDI (PET3)**			
BLT	4.27 ± 3.47^***^	^**^	0.70 ± 1.16
Placebo	2.29 ± 2.69^***^		0.77 ± 1.53
**HAMD (PET1)**			
BLT	11.15 ± 3.80	^**^	0.25 ± 0.45
Placebo	8.91 ± 4.55		0.23 ± 0.60
**HAMD (PET2)**			
BLT	9.23 ± 6.87	^**^	0.5 ± 1.17
Placebo	5.75 ± 4.17		0.14 ± 0.36
**HAMD (PET3)**			
BLT	3.00 ± 3.61	^**,***^	1.10 ± 1.45
Placebo	2.71 ± 2.93		0.23 ± 0.44
**Group size**	24		27
**Age at baseline (mean years±SD)**	34.21 ± 10.57		33.52 ± 10.57
**Sex (male/female)**	8/16		11/16
**Therapy arm (BLT/placebo)**	13/11		12/15

*SAD*: seasonal affective disorder, *HC*: healthy controls, *PET*: positron emission tomography, *BLT*: bright light therapy, *BDI*: Beck Depression Inventory, *HAMD*: Hamilton Depression Rating Scale

^*^Significant difference vs. placebo (*p* < 0.050)

^**^Significant difference vs. HC (*p* < 0.010)

^***^Significant difference vs. PET1 (*p* < 0.050)

Repeated measures ANOVA showed that BDI scores decreased from PET1 to PET2 in the SAD-BLT group (*F*_1, 11.46_ = 7.06, *p* = 0.022) and in the HC-placebo group (*F*_1, 13.12_ = 5.74, *p* = 0.032). Between PET1 and PET3, BDI scores improved in SAD-BLT (*F*_1, 9.53_ = 59.23, *p* < 0.001) and SAD-placebo (*F*_1, 9.60_ = 25.91, *p* = 0.001) groups while HAMD improved in SAD (*F*_1, 21.20_ = 61.97, *p* < 0.001) in general. At PET1, BDI scores differed between SAD-BLT and SAD-placebo, with lower values in the latter (*F*_1, 22.00_ = 4.84, *p* = 0.039). This was no longer the case at PET2, after BLT. SAD showed higher BDI and HAMD scores than HC at all three PET time points (all *p* < 0.010) (Table 1). As BDI and HAMD were, as to be expected, strewn around 0 in the HC group, significant clinical results were confirmed using non-parametric testing (all *p* < 0.050).

Regression analyses showed that global MAO-A V_T_ was not significantly influenced by age, sex, perimenopausal status, nor HAMD scores at PET1, PET2 or PET3. However, BDI had a significant influence on MAO-A V_T_ at PET2 (*R*^2^ = 0.088, adjusted *R*^2^ = 0.067, *p* = 0.045, uncorrected). This association was negative, with lower MAO-A V_T_ related to higher BDI, and was significant when SAD and HC were investigated together. Based on the results of these regression analyses, BDI was included as a covariate in all subsequent mixed model analyses of MAO-A V_T_.

**Fig. 1:**
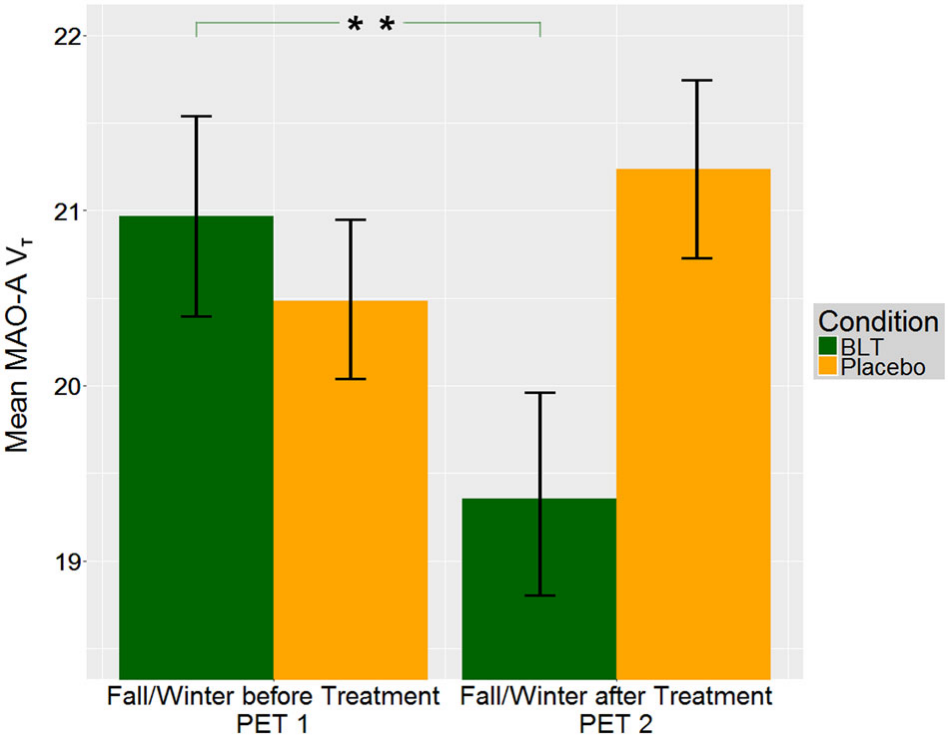
MAO-A V_T_ reduction after BLT. Three weeks of BLT resulted in a reduction in MAO-A V_T_ (*F*_1, 208.92_ = 25.96, *p* < 0.001), while placebo did not. Bars denote mean MAO-A V_T_ for BLT (green, SAD and HC pooled) and placebo (yellow, SAD and HC pooled), brackets denote standard error. Illustrated MAO-A V_T_ is averaged for all ROIs. Effects of BLT and placebo are pooled for HC and SAD because mixed model analyses of PET1 and PET2 data revealed a two-way interaction between measurement and therapy (*F*_1, 385.58_ = 19.16, *p* < 0.001). PET1 fall/winter before BLT/placebo, PET2 fall/winter after BLT/placebo. **p ≤ 0.001. MAO-A V_T_: monoamine oxidase A distribution volume, BLT: bright light therapy, SAD: seasonal affective disorder, HC: healthy controls, 5-HT: Serotonin

**Fig. 2:**
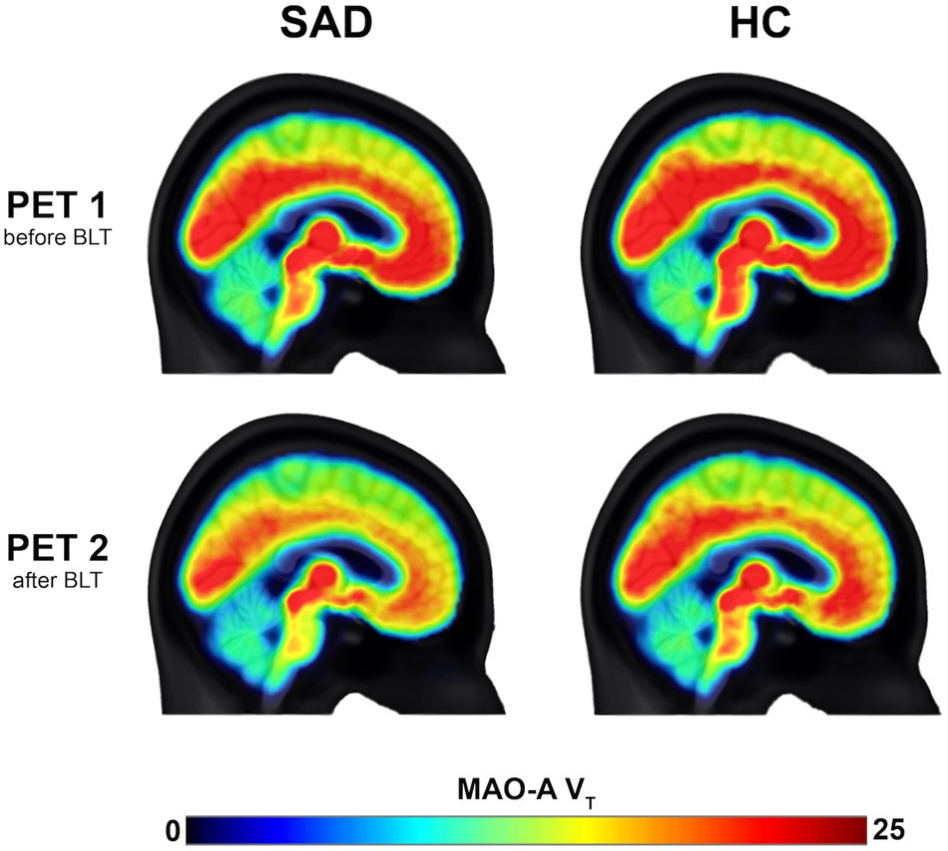
Brain maps of MAO-A V_T_ before and after BLT in SAD and HC. MAO-A V_T_ was reduced significantly after BLT (*F*_1, 208.92_ = 25.96, *p* < 0.001), though not after placebo. MAO-A V_T_ brain maps are shown for SAD (left) and HC (right) before and after BLT. Color bar denotes MAO-A V_T_. MAO-A V_T_: monoamine oxidase A distribution volume, BLT: bright light therapy, SAD: seasonal affective disorder, HC: healthy controls

**Fig. 3:**
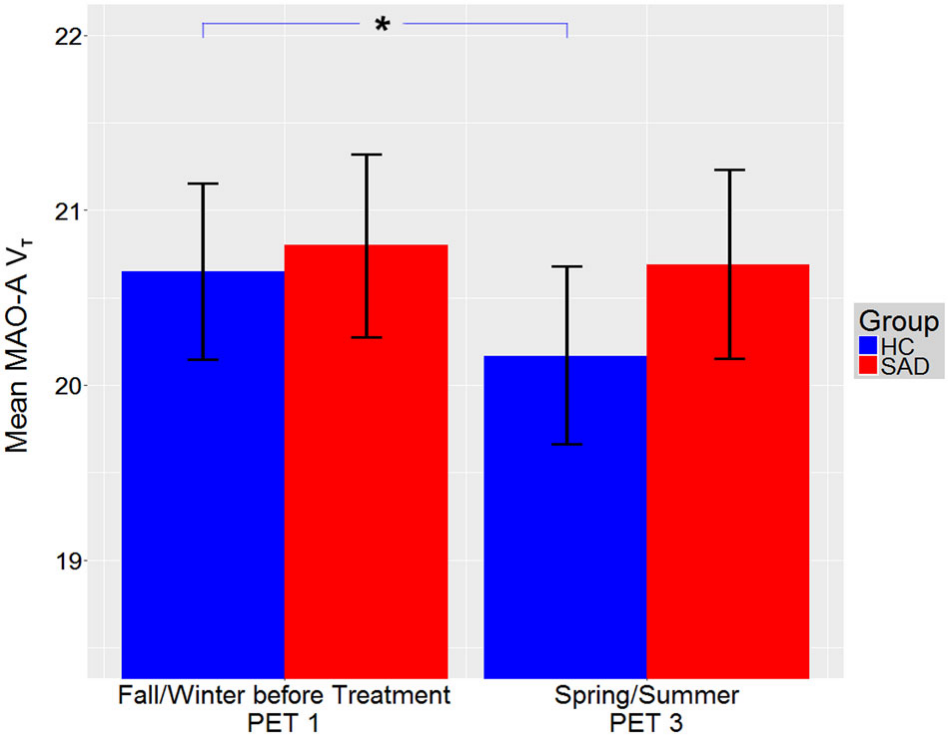
MAO-A V_T_ decrease from fall/winter to spring/summer in HC. MAO-A V_T_ decreased in HC from fall/winter (PET1, before BLT/placebo) to spring/summer (*F*_1, 187.84_ = 4.79, *p* = 0.030), while no change was shown in SAD. Though higher MAO-A V_T_ in HC in fall/winter compared to spring/summer appears contrary to the hyposerotonergic hypothesis of depression, it may be reflective of results from previous studies showing that MAO-A levels are dependent on substrate availability^31^. Higher MAO-A V_T_ in fall/winter may therefore be secondary to other mechanisms that increase serotonin, hereby preventing development of SAD. Bars denote mean MAO-A V_T_ for HC (blue, BLT and placebo pooled) and SAD (red, BLT and placebo pooled), brackets denote standard error. Illustrated MAO-A V_T_ is averaged for all ROIs. *p ≤ 0.050. MAO-A V_T_: monoamine oxidase A distribution volume, BLT: bright light therapy, SAD: seasonal affective disorder, HC: healthy controls

Mixed model analysis of MAO-A V_T_ data from all three time points revealed a three-way interaction between measurement, therapy and group (*F*_2, 555.84_ = 4.28, *p* = 0.014). Between PET1 and PET2, a two-way interaction between measurement and therapy was shown (*F*_1, 385.58_ = 19.16, *p* < 0.001). MAO-A V_T_ fell significantly after BLT (*F*_1, 208.92_ = 25.96, *p* < 0.001) and no change was shown after placebo (Figs. 1 and 2). Between PET1 and PET3 a three-way interaction between measurement, therapy, and group (*F*_1, 341.23_ = 6.46, *p* = 0.011) was shown. MAO-A V_T_ decreased significantly in HC (*F*_1, 187.84_ = 4.79, *p* = 0.030) from fall/winter before treatment (PET1) to spring/summer (PET3), while SAD did not differ in MAO-A V_T_ between these time points (Fig. 3). MAO-A V_T_ did not differ between group (SAD, HC) or therapy (BLT, placebo) at any of the three PET time points. For average regional MAO-A V_T_ see Table 2.

**Table 2:** Monoamine oxidase A distribution volume

ROI	SAD	HC
PET1 (fall/winter before BLT/placebo)		
Frontal pole	18.65 ± 4.49	18.61 ± 4.05
Temporal pole	18.58 ± 4.04	17.81 ± 3.82
Anterior cingulate gyrus	21.48 ± 4.76	21.08 ± 4.94
Posterior cingulate gyrus	21.30 ± 5.14	21.30 ± 4.52
Thalamus	24.58 ± 5.88	25.21 ± 6.35
Caudate	15.67 ± 3.95	15.44 ± 3.15
Putamen	21.36 ± 5.15	21.06 ± 4.55
Hippocampus	20.96 ± 5.02	20.70 ± 4.60
Midbrain	24.71 ± 5.79	24.39 ± 6.05
PET2 (fall/winter after BLT)		
Frontal pole	16.81 ± 5.14	18.00 ± 5.13
Temporal pole	16.99 ± 5.44	17.25 ± 5.33
Anterior cingulate gyrus	19.65 ± 5.97	20.04 ± 6.56
Posterior cingulate gyrus	19.47 ± 6.22	20.07 ± 6.36
Thalamus	22.46 ± 6.88	23.84 ± 6.02
Caudate	14.39 ± 4.84	14.42 ± 3.94
Putamen	19.69 ± 5.97	20.44 ± 5.76
Hippocampus	18.95 ± 6.00	19.80 ± 5.69
Midbrain	22.49 ± 6.16	23.66 ± 7.17
PET2 (fall/winter after placebo)		
Frontal pole	18.89 ± 4.17	19.13 ± 3.22
Temporal pole	18.33 ± 3.63	18.45 ± 3.77
Anterior cingulate gyrus	21.62 ± 4.60	22.58 ± 4.63
Posterior cingulate gyrus	21.50 ± 4.57	22.75 ± 4.08
Thalamus	24.79 ± 5.37	26.19 ± 5.01
Caudate	16.07 ± 4.07	16.36 ± 2.86
Putamen	21.10 ± 4.10	21.82 ± 4.08
Hippocampus	21.15 ± 4.42	21.73 ± 3.92
Midbrain	24.67 ± 5.18	25.12 ± 5.26
PET3 (spring/summer)		
Frontal pole	18.67 ± 3.50	18.23 ± 3.99
Temporal pole	18.85 ± 3.73	17.63 ± 4.16
Anterior cingulate gyrus	22.33 ± 4.95	21.14 ± 5.41
Posterior cingulate gyrus	21.81 ± 4.31	20.88 ± 4.91
Thalamus	23.73 ± 4.71	24.01 ± 4.71
Caudate	14.68 ± 2.55	14.47 ± 3.14
Putamen	21.01 ± 3.77	20.44 ± 4.33
Hippocampus	20.41 ± 3.39	20.18 ± 3.94
Midbrain	25.36 ± 5.35	24.70 ±6.05

Monoamine oxidase A distribution volume (MAO-A V_T_) reported as mean ± SD. *ROI*: region of interest, *SAD*: seasonal affective disorder, *HC*: healthy controls, *BLT*: bright light therapy, *PET*: positron emission tomography

In mixed model analyses excluding subjects with overlapping scan dates (1 SAD, 2 HC) MAO-A V_T_ also increased in SAD-BLT (*F*_1, 84.63_ = 22.60, *p* < 0.001) from PET1 to PET3. All other effects were unchanged from the main analyses.

When performed without BDI as a covariate, mixed model analysis of MAO-A V_T_ data from all three time points revealed a two-way interaction between measurement and therapy (*F*_2, 556.22_ = 14.57, *p* < 0.001) as well as measurement and group (*F*_2, 556.22_ = 10.84, *p* < 0.001), rather than a 3-way measurement by therapy by group interaction. All other effects were unchanged from the main analyses. Mixed model analyses utilizing the difference in MAO-A V_T_ between PET1 and PET3 as dependent variable revealed no significant differences between group or therapy arm in change to MAO-A V_T_ between PET1 and PET3.

## Discussion

In our study, cerebral MAO-A V_T_ did not differ between SAD and HC in fall/winter before treatment, fall/winter after treatment, or in spring/summer. However, cerebral MAO-A V_T_ decreased in HC from fall/winter (before treatment) to spring/summer, while it remained unchanged in SAD. In addition, this is the first study to demonstrate reduction of cerebral MAO-A V_T_ after BLT, in vivo.

Our study was motivated by experiments showing increased MAO-A levels in non-seasonally depressed patients. These findings are a fundament of the hyposerotonergic hypothesis of depression^4,6^. Furthermore, extensive evidence demonstrates that the human serotonergic system is affected by season^8,11^ and light^7,9,10^. Seasonality of symptoms in SAD has been linked to the influence of light^27^ which is underscored by the efficacy of BLT^3^. We therefore hypothesized that the MAO-A may serve as an endophenotypic link between light, SAD symptoms and their treatment.

However, we did not find a difference in MAO-A V_T_ between SAD and HC in fall/winter before (PET1) or after BLT treatment (PET2), or in spring/summer (PET3). On the one hand, our findings suggest that MAO-A V_T_ is not altered in SAD. This explanation would be in accordance with previous studies that show unaltered MAO-A V_T_ in non-seasonal MDD. However, comparability with these earlier studies is limited because they were performed in other brain regions and after suicide^28^. Furthermore, our patient group showed mild typical depressive symptoms, as is often the case in SAD. Therefore, the lack of a statistically significant difference in MAO-A V_T_ between SAD and HC, in contrast to studies in MDD^4^, may reflect the observation that typical depressive symptoms are less severe in SAD than in non-seasonal depression^29^. Studies linking more pronounced symptom severity to higher MAO-A in MDD support this concept^30^.

Our results suggest that changes to MAO-A V_T_ in SAD may instead be based on dysregulation of seasonal changes to cerebral expression levels. Although SAD and HC did not show cross-sectional differences in MAO-A V_T_, HC showed a decrease in MAO-A V_T_ from fall/winter to spring/summer, while SAD did not. Our finding that HC, but not SAD, showed a seasonal dynamic is in line with a previous study showing SERT downregulation in fall/winter in HC, but not in SAD^12^. However, these results are in contrast with another study, which instead showed increased seasonal SERT variation in SAD in comparison to healthy individuals^13^. In our study, while HC showed seasonal change and SAD did not, the seasonal (fall/winter to spring/summer) difference in MAO-A V_T_ did not differ between SAD and HC. In summary, these results suggest that the serotonergic pathology in SAD might rather be understood as altered dynamic regulation of expression, rather than a static change, which is in line with previous studies^12,13^.

In the sub-analysis without the subjects responsible for the seasonal overlap between fall/winter after treatment (PET2) and spring/summer (PET3), the SAD-BLT group also showed seasonal changes. In this group, MAO-A V_T_ increased from fall/winter before treatment (PET1) to spring/summer (PET3). Therefore, the change in MAO-A V_T_ was in the opposite direction as in the HC group. On the one hand, this effect might be understood as an overcompensation, after levels of the enzyme were decreased by BLT. Alternatively, this finding may reflect that BLT treatment activates dynamic regulation of MAO-A V_T_ expression.

HC showed higher MAO-A V_T_ in fall/winter than in spring/summer. Increased serotonergic degradation in healthy individuals appears contrary to previous studies showing increased MAO-A V_T_ in depression. However, this pattern may be reflective of findings showing that MAO-A levels are dependent on availability of 5-HT and other substrates such as dopamine^31^. MAO-A levels were decreased after reduction of 5-HT via tryptophan depletion and increased after application of carbidopa–levodopa^31^. Based on the monoaminergic hypothesis of depression, 5-HT and other monoamine levels may be low in SAD, but not in HC^6^. Therefore, other mechanisms in HC may in fact elevate, or prevent reduction of, 5-HT^12^, though these regulatory processes are likely to be complex^32^. In theory, this would result in an increase in MAO-A V_T_^31^. In summary, the increased MAO-A V_T_ seen in HC in fall/winter in comparison to spring/summer might reflect that seasonal MAO-A V_T_ changes are secondary to other changes within the serotonergic system.

MAO-A V_T_ decreased after BLT. Lower MAO-A V_T_ would in theory result in a reduction in 5-HT degradation^5^, resulting in higher 5-HT levels and ultimately antidepressant effects. This is in analogy to the effects of monoamine oxidase inhibitors^33^ and other serotonergic antidepressants such as selective serotonin reuptake inhibitors^6^. MAO-A V_T_ reduction was not shown after placebo, thus demonstrating that the effect is specific to BLT and not merely an effect of time. However, downregulation of MAO-A V_T_ after BLT was not specific to the SAD group, as demonstrated by the measurement by therapy, rather than measurement by therapy by group interaction, between PET1 and PET2. Therefore, we suggest that reduction of MAO-A V_T_ may be understood as an unspecific neurobiological correlate of intense light exposure, in this case via BLT, because the effect was not specific to underlying pathology.

Several limitations must be discussed. The BDI and the 24-item HAMD are beneficial as they encompass self- and external ratings. However, although they do assess some atypical depressive symptoms such as fatigue, not all are addressed. This factor may explain why we did not find a more pronounced association between symptom severity and MAO-A V_T_ in SAD, particularly as MAO-A levels have previously been linked to atypical depressive symptoms^30^. Furthermore, BDI differed between the SAD-placebo and SAD-BLT arms before treatment. However, this point was assessed via inclusion of BDI as a covariate in the subsequent mixed model analyses of MAO-A V_T_. HC showed particularly low BDI and HAMD scores. As a result, the effect of BLT and season on the HC’s BDI scores may not be adequately assessed while changes to BDI and HAMD in SAD may be overestimated. For placebo treatment, a light intensity of 400 lux was chosen because this intensity was previously described as not biologically active^22^, albeit similar enough to verum BLT (10,000 lux) so as not to unblind treatment. However, we cannot exclude the possibility that this low intensity light has some therapeutic effect. Therefore, the difference in MAO-A V_T_ between BLT and placebo may in fact be more pronounced. Another potential limitation is the minor overlap between the fall/winter and spring/summer seasons. This point was addressed through a sub-analysis which excluded affected subjects and replicated original results. In addition, though it is a widely utilized method, implemented in PMOD (PMOD Technologies Ltd., Zurich, Switzerland; www.pmod.com), the Logan plot may underestimate MAO-A V_T_ at the voxel level^34^, an effect which is dependent on signal noise level and the extent of the V_T_^35^. However, potential bias would be circumvented by the fact that MAO-A V_T_ was quantified utilizing the same methodology in all subjects.

In addition, if we assume that differences in MAO-A V_T_ between SAD and HC are discrete, in accordance with the concept of SAD as a less severe sub-set of MDD, the subject size may be considered a limitation, though it is similar to previous [^11^C]harmine PET studies^4^. Along this line, the effects shown in our study were statistically significant when all regions were investigated together, but not on the individual region level. This finding may be a result of discrete differences in MAO-A V_T_ within individual regions that are elucidated when statistical power is increased by joint evaluation of all regions.

In order to address potential variability and reduce bias in our sample, numerous clinical variables including age, sex, symptom severity, perimenopausal status and smoking^20^ were corrected for, either through regression analyses (age, sex, symptom severity, perimenopausal status) and subsequent inclusion as a covariate when significant (this was only the case for BDI), or through definition as an exclusion criterion (smoking). The influence of BLT on MAO-A V_T_, yet not placebo, as well as the seasonal effects in HC, yet not SAD, were also significant in an exploratory analysis in which BDI was not included as a covariate. This suggests that the influence of BDI on MAO-A V_T_ that was elucidated in regression analysis is likely discrete. In addition, it emphasizes that the influence of BLT and the seasonal effects in HC are likely specific effects of light and season, and not directly related to symptom severity. Previous studies illustrate a lack of an influence of sex on MAO-A V_T_^4^, as was the case in our study. However, the MAO-A gene is encoded on the X-chromosome^36^ and an influence of estrogen^37^ and testosterone^38^ on MAO-A expression has been demonstrated. Increased MAO-A has been shown in perimenopause, a phase which is characterized by a drop in estrogen levels, which has been linked to depressive symptoms^37^. Though we corrected for various clinical factors, it is possible that the factors discussed above, such as sex, exert influence on MAO-A V_T_ through interaction with other clinical variables that were not assessed. We base this assumption on studies which demonstrate a gender specific influence of MAO-A genotype on various clinical outcomes^39,40^. In addition, genetic variables that are not accounted for in this study may play a role. For example, an influence of genetics on how the serotonergic system is influenced by light has been demonstrated with the 5-HTTLPR^41,42^. An influence of MAO-A genotype on propensity for seasonal variation in protein expression has to our knowledge yet to be shown, but cannot be excluded. Furthermore, various components of the serotonergic system demonstrate diurnal patterns of variation^11^. In fact, circadian changes to MAO-A activity have been demonstrated in animal studies^43^. Therefore, it must be considered a limitation that not all PET measurements took place during the same time of day. However, time of day of PET measurement varied within groups (SAD, HC), treatment-arms (BLT, placebo) and measurement (PET1, PET2, PET3), which likely mitigates a potential effect of this factor on our results.

In summary, we did not find differences in cerebral MAO-A V_T_ between SAD patients and controls when our data was assessed cross-sectionally, i.e., SAD and HC did not differ in fall/winter before treatment, after treatment or in spring/summer. The lack of a difference in MAO-A V_T_ between these two groups, which is in contrast with previous studies in non-seasonal depression, may result from lower symptom severity in SAD in comparison to MDD^29^. However, HC showed a seasonal dynamic to MAO-A expression, which was not shown by SAD patients. This suggests that SAD may lack seasonal dynamics in brain MAO-A levels. This pattern is consistent with that shown in previous studies on the SERT in SAD^12^. We also demonstrate that BLT results in reduction in MAO-A V_T_, which is likely to result in an increase of 5-HT levels^5^, and is thus in accordance with the serotonergic hypothesis of depression and antidepressant treatment. However, this effect was not specific to the patient group, suggesting that these results reflect a general, neurobiological effect of BLT on the serotonergic system rather than a specific treatment effect.

## Electronic supplementary material

Supplement
