# Supplementary material for: Brain monoamine oxidase A in seasonal affective disorder and treatment with bright light therapy
Source: Transl Psychiatry. 2018 Sep 21;8:198. doi: 10.1038/s41398-018-0227-2 (PMC6155094; doi:10.1038/s41398-018-0227-2)
Supplement: Supplementary file 1 — Supplement [file 41398_2018_227_MOESM1_ESM.docx]

**Supplement**


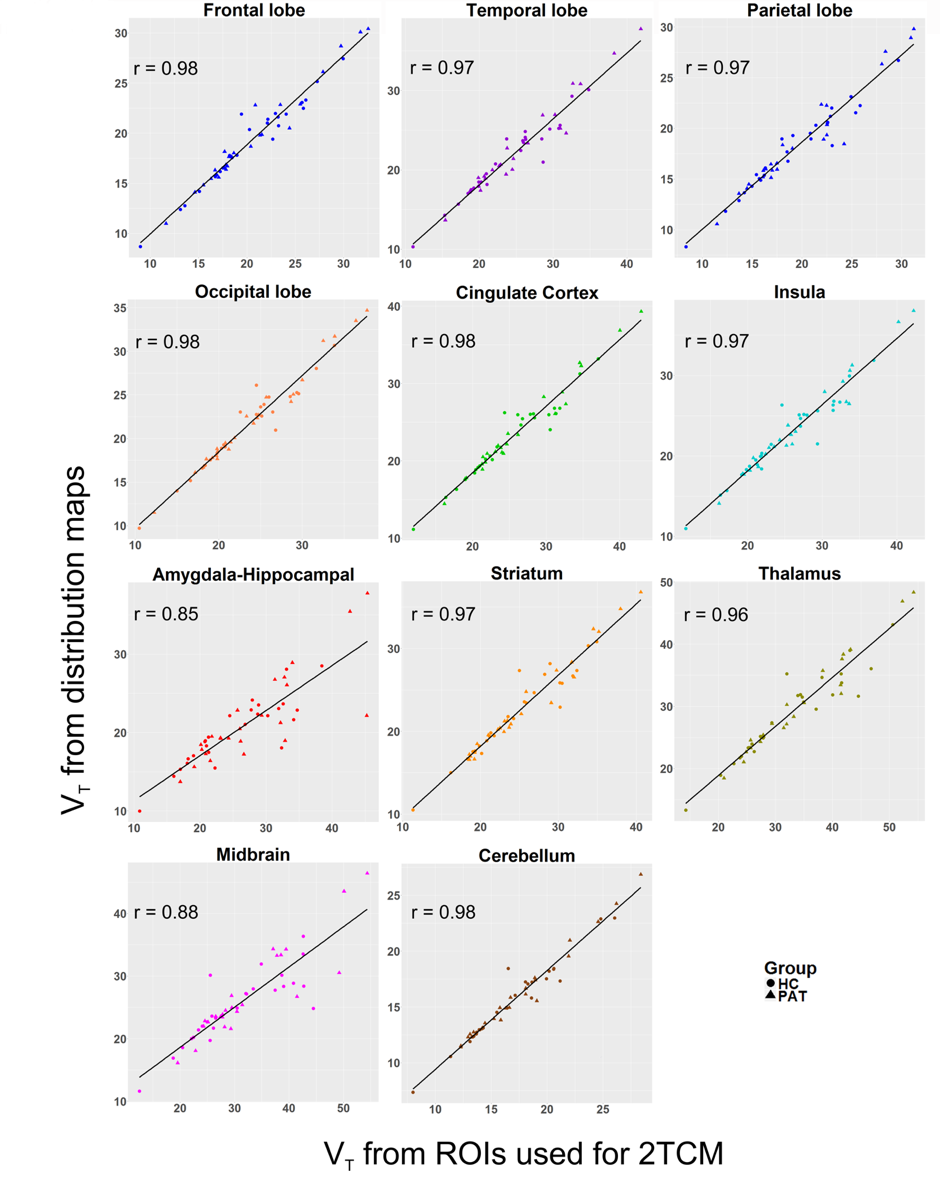


**Figure S1:**

This figure shows the relation of V_T_ values from the ROIs used in coupling of K1/k2, which were calculated using the time activity curve of the entire region (x axis) and V_T_ values used in statistical analysis, which were extracted from distribution images quantified using Logan plot and averaged for all voxels within the ROI (y axis). This analysis was performed in order to demonstrate consistency of the quantification methods.

HC: Healthy controls, Pat: Patients with seasonal affective disorder, V_T_: Distribution volume

**Repeated measures ANOVA of K1/k2 and free fraction of tracer in blood**

Separate repeated measures ANOVA were performed with K1/k2 values and free fraction (f_P_) values of tracer in blood with K1/k2 and f_P_ as dependent variables and measurement (PET1, PET2, PET3) as within subject factor. No significant main effects of measurement were found with either dependent variable, i.e. K1/k2 and f_P_ did not change significantly over time. This demonstrates that changes shown in V_T_ in the main analyses can be attributed to changes in k3/k4.
